# Advances in the Development of Anti-Adhesive Biomaterials for Tendon Repair Treatment

**DOI:** 10.1007/s13770-020-00300-5

**Published:** 2020-11-04

**Authors:** Haiying Zhou, Hui Lu

**Affiliations:** Department of Orthopedics, The First Affiliated Hospital, College of Medicine, Zhejiang University, #79 Qingchun Road, Hangzhou, 310003 Zhejiang Province People’s Republic of China

**Keywords:** Tendon injuries, Tissue adhesion, Biocompatible materials, Hydrogels, Electrospun fiber membrane

## Abstract

**Background:**

Peritendinous adhesion that simultaneous with tendon healing link the healing tendon to the surrounding tissue. It results in functional disability, and has a significant adverse impact on health as well as social and economic development.

**Methods:**

Based on a search in the PubMed and Web of Science database, the research articles were screened by their time, main idea, impact factor index, while the ones with no credibility were excluded. Afterwards, we go through the analysis of the reliability and characteristics of the results were further screened from selected articles.

**Results:**

A total of 17 biomaterials used to evaluate the adhesion mechanism and the properties of the material were found. All of these biomaterials contained randomized controlled studies and detailed descriptions of surgical treatment that support the reliability of their results which indicates that biomaterials act as barriers to prevent the formation of adhesion, and most of them exhibit satisfactory biocompatibility, biodegradability or selective permeability. Moreover, a few had certain mechanical strength, anti-inflammatory, or carrier capacities. However, there still existed some defects, such as time, technology, clinical trials, material targeting and different measurement standards which also lowered the reliability of their results.

**Conclusion:**

In future, anti-adhesion biomaterials should focus on affordable raw materials with wide sources, and the production process should be simplified, in this way, the versatility and targeting of materials will be improved.

## Introduction

Peritendinous adhesions are fibrous adhesions between the tendon and the surrounding tissues caused by collagen deposition that is triggered by the healing process of tendon injury. Tendon injury is the most common soft tissue injury in the field of orthopedics [1]. Studies have showed that 7–15% of patients after tendon repair have complications such as scar formation, increased tendon adhesions and limited joint movement in the early healing stage. Among these patients up to 10% of them will require secondary operations. Progressive peritendinous adhesions will bind the healed tendon to the surrounding tissue, leading to functional disability and eventually resulting in the decreased work ability and high costs [2].

**Fig. 1:**
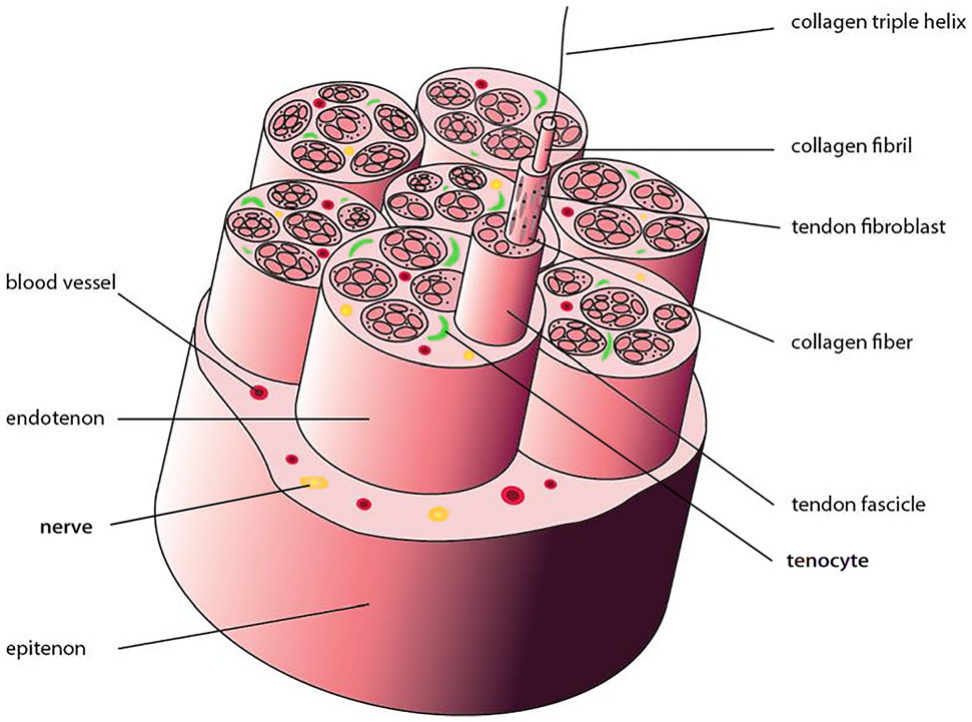
Tendon hierarchy: collagen fibers dominate, tenocytes, nerves and blood vessels are scattered. Adapted with permission from Yang et al. [9]

Tendon adhesions were once thought to be an integral part of tendon healing [3], but better understanding of tendon biology has revealed that not to be the case. Although the pathophysiological mechanism of its occurrence remains undefined, it is generally accepted that the formation of adhesion is related to the superiority of external tendon healing. More specifically, the intrinsic healing via proliferation of epitenon and endotenon tenoblasts contributes to better biomechanics and less dysfunctions, while extrinsically healed by invasion of cells from surrounding sheath and synovia, it facilitates adhesion formation thereby damaging the tendon gliding [4, 5]. Besides, the release of cytokines and recruitment of fibroblasts in the early inflammatory response after tendon injury greatly promotes adhesion [6]. Meanwhile, the repair of tendon tissue is limited by its inherent low cellularity and metabolic activity coupled with limited blood and nerve supply, leading to a slower healing rate than many other tissues [7] (Fig. 1).

Based on research regarding the mechanism of tendon adhesion, scientists have explored many advanced treatment methods, including surgical and non-surgical options [8], to inhibit exogenous healing, promote endogenous healing, reduce inflammation, and eventually restore the function of tendon sliding totally [9]. These strategies can be broadly classified into four categories: intraoperative prevention, surgical methods, use of physical barriers, and use of chemical agents [10]. Among them, use of physical barriers can control the factors that adversely affect tendon healing. Since Henze and Mayers applied silk to prevent tendon adhesion *in vitro*, anti-adhesion materials have experienced great improvement in the last 100 years. Especially in the past 30 years, the emergence of bionic materials and degradable polymers has attracted worldwide attention for the applicability and functional development of anti-adhesion materials. However, our review of articles revealed that due to limitations such as high production costs and manufacturing processes, the development of most of the materials is still at the animal testing stage.

Therefore, in this review, we focus on the application of several advanced synthetic materials used to prevent tendon adhesion in the past 10 years. By analyzing their composition and synthesis principles, the anti-adhesion mechanism and effect can be clearly expounded. Even though the optimum form of anti-adhesion materials has not been agreed on yet [1, 11], we hope that by comparing current anti-adhesion studies and analyzing the existing problems and defects, we can provide researchers with a promising prospect.

## Methods

Guidance from the Preferred Reporting Items for Systematic Reviews and Meta-analyses (PRISMA statement) was followed in this paper. This study is based on the analysis and comparison of popular and/or novel anti adhesion agents used in biomaterials in the past decade.

### Database searches

We found most of the information in PubMed database and a few studies in Web of Science by using the keywords ‘biomaterials’ and ‘tendon adhesion’.

### Study assessment

Studies were selected based year of publication (last 10 years), impact factor index (in case of multiple publications on a topic, the journal with higher impact factor was chosen), major point (only articles related tendon and anti-adhesion materials were chosen), innovation, practicality, and credibility. We then analysed the characteristics of the research conducted in the article, finding their potential effect modifiers, reasons for heterogeneity and deficiencies in the implementation of the study, which might inspire the improvement of future research.

The materials were divided into three main categories according to their structure: hydrogel, fibrous membrane and absorbable film. The characteristics of the materials in the groups were analyzed, and the advantages and disadvantages of each category were summarized. Afterwards, the comparison among different groups was made, and the characteristics, existing research gaps and possible development directions of the anti-adhesion biomaterial were identified.

## Results

### Searching results

After searching in PubMed and Web of Science databases, a total of 47 articles were selected, including 17 that described biomaterials with effective anti-adhesion outcomes that were representative of the three kinds of anti-tendon adhesion materials, eight articles were about the mechanism of tendon healing, five articles were about tendon injury, and the rest were about raw materials, failed or duplicated material and other relevant topics.

### Study assessment

Since most of the biomaterials described in the current study involve research at animal experimental stage (Table 1), there was almost no follow-up bias. Besides, all articles had randomized controlled studies and enough detailed descriptions of surgical treatment. However, some heterogeneity could result from other factors that have potential impact on experimental results. Such factors include differences in animal models, experimental methods, techniques of experimental operators, settings of control groups, methods of result analysis and observers, of which Tang’s adhesion assessment system was one of the most commonly used (Table 2). Besides, some of the studies had not conducted double-blind experiment, which might have led to a certain detection bias in the results. Meanwhile the duration of the researches was too short with 10 subjects having been conducted in only 3 weeks, which might miss the long-term effect of biomaterials on tendon healing. As studies with positive results are more likely to be published, when we analyzed the experimental results of various materials, there was a certain publication bias and reporting bias.

**Table 1:** The characteristics of each biomaterial

Materials	Timespan	Study size	Subject	Outcome criteria	Blinded evaluator
Seprafilm	8 weeks	40	Chicken	ASS^1^ described by [12]	No
Xanthan gum/gellan gum/hyaluronan hydrogel membranes	3 weeks	18	Rats	ASS described by [13]	Yes
PMBV^2^	3 weeks	90	Rats and Rabbits	ME^3^ described by [14] HE^4^ described by [12]	Yes
PMA^5^/PMB^6^/Fe3+ hydrogel	3 weeks	43	Rats and Chicken	ASS described by [15]	Yes
Thermo-responsive in-situ forming hydrogels	3 weeks	40	Rabbits	Comparison between experimental data	No
Hydrogel with 5-FU^7^	3 weeks	30	Chicken	ASS described by [12]	No
DegraPol tube	6 weeks	30	Rabbits	ASS described by [12]	Yes
MMC-PLLA^8^	3 weeks	126	Rats and Rabbits	ASS described by [15]	Yes
ES/PTB^9^	4 weeks	24	Rabbits	ASS described by [16]	Yes
HA/PCL-PCL^10^	3 weeks	24	Chicken	ME described by [15] HE described by [12]	Yes
PCL-g-CS NFM^11^	8 weeks	96	Rabbits	Comparison between experimental data	No
HA/ibuprofen core/PEG/PCL/Ag shell^12^	3 weeks	32	Rabbits	ASS described by [17]	No
Reconstituted type I collagen membrane [18]	3 weeks	18	Chicken	ASS described by [19]	Yes
Integra	3 weeks	32	Chicken	Customized ASS	Yes
Decellularized amnion	12 weeks	60	Chicken	ASS described by [12]	No
P(LA/CL) membrane^13^	4 weeks	142	Rabbits	ME described by [20]	No
WBPU^14^	4 weeks	30	Rabbits	Customized ASS	Yes

^1^ ASS: adhesion-scoring system, ^2^ PMBV: an MPC hydrogels consisting of poly(2-methacryloyloxyethyl phosphorylcholine-co-nbutyl methacrylate-co-p-vinylphenylboronic acid)(PMBV) and poly(vinyl alcohol)(PVA), ^3^ ME: macroscopic evaluation, ^4^ HE: histological evaluation, ^5^ PMA: poly(MPC-co-methacrylic acid), ^6^ PMB: poly(MPC-co-n-butyl methacrylate), ^7^ 5-FU: 5-fluorouracil, ^8^ MMC-PLLA: mitomycin-C loaded poly(l-lactic acid), ^9^ ES/PTB: electrospun silk stained with a photochemical tissue bonding dye, ^10^ HA/PCL-PCL: a biomimetic bilayer sheath membrane consist of the inner layer which is HA loaded poly(ε-caprolactone) (HA/PCL) and the outer PCL membrane, ^11^ PCL-g-CS NFM: electrospun chitosan-grafted polycaprolactone nanofibrous membrane, ^12^ HA/ibuprofen core/PEG/PCL/Ag shell: core–shell nanofibrous membranes consist of the poly(ethylene glycol)(PEG)/PCL shell which contains silver nanoparticles(Ag NPs) and HA/ibuprofen core. ^13^ P(LA/CL) membrane: poly l-lactide-co-ε-caprolactone membrane, ^14^ WBPU: waterborne biodegradable polyurethane films

**Table 2:** The adhesion-scoring system [12]

*Adhesion macroscopic evaluation criteria*		
Criteria	Point	Adhesion appearance
Length	0	No adhesion
	1	Localized, < 10 mm longitudinal
	2	10–15 mm
	3	Intense, > 15 mm
Characteristics	0	No adhesion
	1	Loose, elastic, and mobile
	2	Of average thickness and mobile
	3	Thick, hard, and immobile
Grading	0	No adhesion
	1	Mild adhesion
	2	Moderate adhesion
	3	Advanced stage adhesion

*Histopathological evaluation criteria*		
No adhesion	0	Normal
Light degree adhesion (good)	1	Few flaments. Fine, long flament structure
Moderate adhesion	2	Average number of flaments. Large, thick flament structure
Severe adhesion	3	Loss of flament structure. Dense fbrosis

### Assessment of biomaterials

#### Hydrogels

**Fig. 2:**
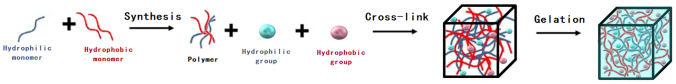
The process of hydrogel formation. Adapted with permission from Thoniyot P, Tan MJ, Karim AA, Young DJ, Loh XJ. Nanoparticle-hydrogel composites: concept, design, and applications of these promising, multi-functional materials. Adv Sci (Weinh). 2015;2(1–2):1400010

Hydrogels are polymers with large water content. Their three-dimensional structure shows hydrophobic groups and hydrophilic residues that are scattered in the crosslinking structure of the water-soluble polymer. In this structure, hydrophilic residues are wrapped in the inner part after combining with hydrogen whereas hydrophobic groups distributed in the outer part become swollen when encountering water [21] (Fig. 2). This specific structure enables it to slowly release the internal material by adjusting the rate of decomposition of the degradable external hydrophobic groups [22]. Besides, it can simulate the extracellular matrix (ECM) of the tendon thus has good biocompatibility. In addition, its stress relaxation properties can enhance the osteogenic differentiation of mesenchymal stem cells (MSCs) [23]. By adjusting the concentration and crosslinking levels, one can obtain appropriate mechanical properties and optimize the degradation rate, which eliminates the need for surgical intervention after healing [21]. Meanwhile, after crosslinking, hydrogels can either become stable or sensitive temperature or pH. Besides, its nanostructure can inhibit fibroblast migration and isolate the injured tissue from a strong immune response. It also allows the penetration of oxygen, nutrients and growth factors that are important for tendon healing. Furthermore, its mobility enables it to cover small or irregular wounds, accommodate endoscopic techniques, and does not put a strain on the narrow tendon-sheath tunnel [1].

##### Hyaluronic acid hydrogels

Hyaluronic acid (HA) is a non-specific hydrophilic polysaccharide existing in synovial fluid. It is an essential source of nutrients and lubricant for tendon gliding. Being a high molecular weight (MW) material, it can take up larger space to perform a barrier and act as a placeholder to reduce local bleeding. It also has anti-inflammatory properties by inhibiting harmful proinflammatory factors [24]. Additionally, it has superior viscoelasticity, moisture retention and its inherently disordered network structure can separate wound surfaces and reduce friction. Moreover, because of its negative charge, HA can inhibit the proliferation and migration of fibroblasts, thereby reducing adhesion. Since the confirmation of its anti-adhesion effect in the rabbit flexor tendon rupture model in 1986 [25], hundreds of biomaterials of HA and its derivatives have been created and placed into trials and clinical applications.

For instance, Seprafilm [26] consists of two anionic polysaccharides, HA and Carboxymethylcellulose (CMC) It has been observed to combine inhibition effect on fibroblastic activity, anti-inflammatory effect from CMC and healing promotion properties from HA. As a composite material, it has additional superiority; for example, no cases of necrosis or infection of tendon body has been reported, which shows that Seprafilm has no negative effect on tendon repair and does not support the bacteria infections. Moreover, it is non-reactive and can continue to function in the presence of blood.

However, in clinical practice, Seprafilm degrades in 7 days, which is too short for a time for tendon healing. In addition, the high cost, lack of deformability, difficultly in wrapping around the tendon well, and the inability of the barriers to function in certain circumstances such as infection post-anastomosis, adversely limit the use of these products. Thus, Kuo et al. [27] reported a study of xanthan gum (XG)/gellan gum(GA)/hyaluronan (XGH) hydrogel membranes that was more practical. Both XG and GA are low cost and easily produced. XG is a polysaccharide secreted by the bacterium *Xanthomonas campestris* and is hydrocolloids. It is used to moderate the characteristics of XGH membranes to quickly swell and soften after immersion in phosphate-buffered solution (PBS) for 3 min. This property facilitates the clinical manageability and use in surgical procedures. GA is a linear, anionic polysaccharide secreted by *Pseudomonas elodea*. During tendon repair it can remain at a repair site for several weeks, which is a major advantage of using this material. Additionally, recent discoveries of GA as a drug carrier also opens up the prospect of such materials. Concurrently, XGH is as effectively as Seprafilm and can reduce tendon adhesion and enable tendon healing without weakening the mechanical strength of healing tendon.

##### Polymer of 2-methacryloyloxyethyl phosphorylcholine hydrogels

2-Methacryloyloxyethyl phosphorylcholine (MPC) is a type of phospholipid, which means it has good biocompatibility. When compared to other polymers, the higher free water fraction of phospholipid during hydration enables it to reduce protein adsorption without causing protein conformational changes [28]. Meanwhile, the dissociation rate of MPC hydrogels is controllable, and it has good antithrombotic properties. No foreign body reaction or adverse reactions were found in the process of tendon healing [13, 29]. And no subsequent bone resorption reactions were found in orthopedic applications [29].

A polymer of MPC hydrogels [29] formed from two aqueous polymer, one contained MPC called 5.0% poly(2-methacryloyloxyethyl phosphorylcholine-co-nbutyl methacrylate-co-p-vinylphenylboronic acid) (PMBV) and the other was 2.5% poly(vinyl alcohol) (PVA), was proven to have good biocompatibility, no foreign body reaction, and could be used as a physical barrier to reduce tendon adhesion. Furthermore, its degradation rate was controlled by the PMBV concentration, and the experiments *in vivo* and *in vitro* proved that hydrogel with 5% PMBV took more than 3 weeks to degrade, a duration that covers the key stage of tendon healing.

Another spontaneously forming MPC polymer hydrogel [13] was prepared by mixing the aqueous poly(MPC-co-methacrylic acid) (PMA) and amphiphilic poly(MPC-co-n-butyl methacrylate) (PMB), in the presence of Fe^3+^. The rate of degradation of this hydorogel was regulated by ionic crosslinking mechanism, which results from Fe^3+^ and carboxylate anions in this study.

##### Hydrogels cross-linked with functional molecules

By using a number of crosslinking techniques, hydrogels made sensitive to temperature or pH. Hydrogels based on Poly(*N*-isopropylacrylamide) (PNIPAM) contain this well-known thermo-responsive property that manifests as a reversible sol-gel phase transition behavior, which means they are liquid when the temperature is below their lower critical temperature (LCST), approximately 30 °C, and transform into a gel state at higher temperature [30]. This property enables it to change its shape according to clinical needs, so that it can be applied to the lesion more accurately.

When integrated with chitosan (CS) and HA to form a hydrogel called HA-CS-PNIPAM (HACPN) [31], the polymer can regulate the LCST to approximate physiological value, enhance the mechanical strength, and retain biocompatibility. Furthermore, the additives significantly maintained the water content of the polymer after sol–gel transition and decreased the degree of volume shrinkage, which is originally 90% volume shrinkage, as a result of thermo-responsive behavior. This prevents the cells from passing through the gap created by the contraction. Although the non-biodegradable PNIPAM component monomeric *N*-isopropylacrylamide is toxic, it did not show detrimental effects *in vivo*. Under in vivo conditions, CS was predominantly degraded by enzyme [32], whereas the biodegradation of HA is through enzymatic or non-enzymatic reactions [33]. In addition, MW of PNIPAM (21 kDa) used for synthesizing HACPN is below the renal cutoff (~ 40 kDa) and did not affect tendon healing.

Hydrogels can also be combined with medicines to achieve more significant anti-adhesion effect. Several studies have shown that 5-fluorouracil (5-FU) [22] can block the proliferation of fibroblasts which are associated with the extrinsic healing mechanism. When combined with hydrogels, continuous release of the drug during tendon healing process can be achieved, and the possibility of migration from the target area can be reduced. Moreover, the system expands the scope of drug application. However, studies found that the anti-adhesion effect of the materials is influenced by drug dose with local high concentration aggravating adhesion formation. For instance, the outcomes of 10 mg dose application group was similar to that of non-surgical group, while application of 20 mg and 30 mg resulted in severe fibrosis, adhesion formation, histological and biomechanical damage. Therefore, use of hydrogels as drug delivery system to prevent the formation of adhesion merits further study.

In summary, hydrogels have a good anti-adhesion ability as well as modification potential. When cross-linked with a variety of active substances, it may have multiple effects and will have a synergistic anti-adhesion performance. Thus we recommend designing a double or even multi-response gel that could be activated in different healing stages according to temperature, light or the cellular environment at the site of injury. This will lead early initiation of endogenous repair that increases the strength of the tendon and allows patients carry out functional exercise as soon, and inhibit the exogenous repair to reduce adhesion formation.

However, there are still some shortcomings that need further research such as the optimal degradation rate to cover the period of tendon healing and suitable mechanical properties. This indicates that without affecting the range of motion of the healed joint, hydrogels can be used as a scaffold for tendon cell extension in the process of tendon repair with a certain degree of toughness to protect the tendon from rerupturing and provide a proper molding time to ensure that it can wrap around the repaired parts better. Besides, as a solution, hydrogels type barriers are easier to apply but may be washed out readily and render lower efficacy [34].

#### Electrospun fiber membranes

**Fig. 3:**
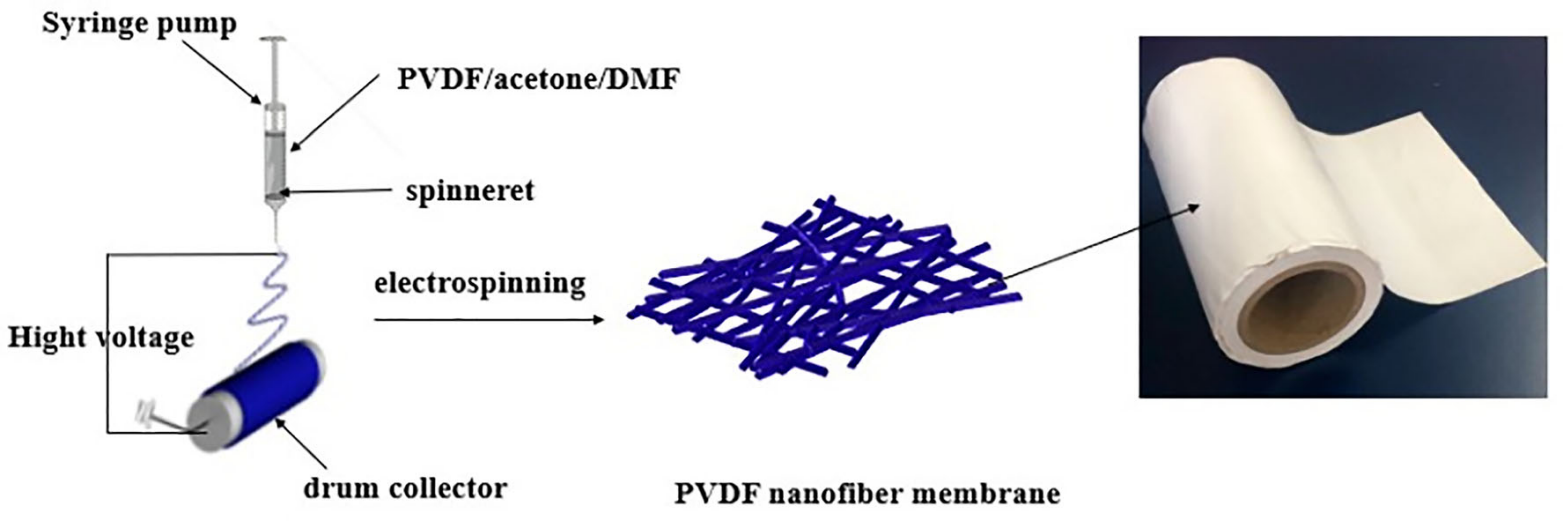
The electrospinning process: First, high voltage static electricity accelerates charged polymer droplets in a capillary Taylor cone vortex and eventually form a jet trickle. The solvent then evaporates in the trickle jet process, and the polymers solidify in the receiving device to form a nonwoven fabric similar to the fiber film. Adapted with permission from Cheng F, Ou Y, Liu G, Zhao L, Dong B, Wang S, et al. Novel quasi-solid-state electrolytes based on electrospun poly(vinylidene fluoride) fiber membranes for highly efficient and stable dye-sensitized solar cells. Nanomaterials (Basel). 2019;9(5)

Electrostatic spinning technology is a method that produces nanofibers through using an electric field [21] (Fig. 3). The fibers can be produced through different technologies, including blending, co-solvent, microsol, emulsion, coaxial and multilayer. With their small diameters, large specific surface area and porosity, nanofibers can simulate the structure and biological functions of tendon ECM, thus can act as a barrier against adhesion without affecting the normal healing. Besides, they take long to degrade *in vivo* than hydrogels, which makes them suitable as a long-lasting anti-adhesion materials [21]. In addition, it has good biocompatibility and limited immune activity, which reduces the incidence of complications and does not adversely affect the tendon healing process. They not only act as scaffold to support cell attachment and proliferation, but also provide a degree of mechanical strength that reduces the tensile load on injured tendon [35]. Furthermore, electrospun fiber membranes can be easily functionalized as active molecule carriers to continuously release drugs or therapeutic cells *in vivo*, thereby attract the attention of many researchers.

According to its structural characteristics, it can be divided into single-layer electrospun fiber membrane and multi-layer electrospun fiber membrane. Although more complicated to make, the multi-layer membrane can more accurately simulate the complex structure of biological tissue and produce materials with different functions and properties to meet the clinical needs.

##### Single-layer electrospun fiber membrane

Burgisser et al. [36] studied the properties of an electrospun DegraPol (DP) tube as an anti-adhesion barrier. DegraPol (DP) is a trade name of a biodegradable polyester polyurethane polymer, synthesized by poly-hydroxybutyrate as crystalline phase and ε-caprolactone as soft phase. It allows extensive degradation half-lives, stabilities, mechanical properties, and biomechanical characteristics by adjusting the ratio of its hard and soft segments. Besides, it has a low inflammatory response, with only a slight increase in lymphocytes and almost no macrophages were found in the study. More importantly, compared with other materials, DP tube implantation did not damage the healed tendon strength or cause a reduction in other biomechanical parameters, such as tensile force, stress and rigidity. In addition, DP degradation is pH-neutral, which does not adversely affect the properties of synovial fluid compared to the acidity in other polymers (like poly(lactide-co-glycolide) (PLGA)).

Due to the ability of electrospun fiber membranes to act as active molecule carriers, Zhao et al. [37] constructed electrospun fibers with a controllable release of mitomycin-C (MMC) by means of microsol electrospinning—the HA hydrogel particles that embeds the MMC were wrapped in poly(l-lactic acid) (PLLA) fibers. MMC can induce the apoptosis of fibroblasts, and can inhibit collagen synthesis in fibroblasts and HaCaT cells [38]. Hence, its application may reduce adhesion formation. However, inappropriate use of MMC may result in acute or chronic toxicity. For example, when local drug concentrations are too high, it can exacerbate scar formation and even wound healing failure. Therefore, based on the water solubility of MMC and the slow-release ability of hydrogel, hydrogel is selected as the best drug carrier. Notably controlled release of MMC not only ensures low dose of local drugs, but also avoids the adverse effect of repeated injection on tissue healing. In addition, as an isolated system, hydrogel can protect its internal MMC activity. However, the fast biodegradation of hydrogel is still a challenge. Therefore, PLLA is used to encapsulate HA hydrogel to prolong the effective time of drug action, for degradation time of PLLA *in vivo* can be extended from weeks to months by varying its MW. At the same time, as HA is released from the PLLA fiber, it can promote tendon healing and lubricate tendon sliding. Through these synergistic actions of different substances, tendon adhesion can be effectively inhibited.

Electrospun silk (ES) can also be modified to become temperature or PH sensitive. When stained with 0.1% Rose Bengal (RB), which is a photochemical tissue bonding (PTB) dye, the ES/PTB becomes a photoactivated nano electrospun fiber film [35]. Irradiation of RB with green light induces photochemical cross-linking with the application site to seal the wound surface. This enhances the early postoperative healing. When tissues are tightly bound to the ES/RB surface, the photochemical cross-linking process is limited to a narrow two-layer space, thus increasing the strength and action of the bond. Because the raw materials of ES are similar to collagen fibers, ES/PTB is also biocompatible and has a low incidence of complications [39]. Overall, this new biomaterial provide a strong support to early wound healing that promotes tendon healing and reduces adhesion formation.

##### Multi-layer electrospun fiber membrane

**Fig. 4:**
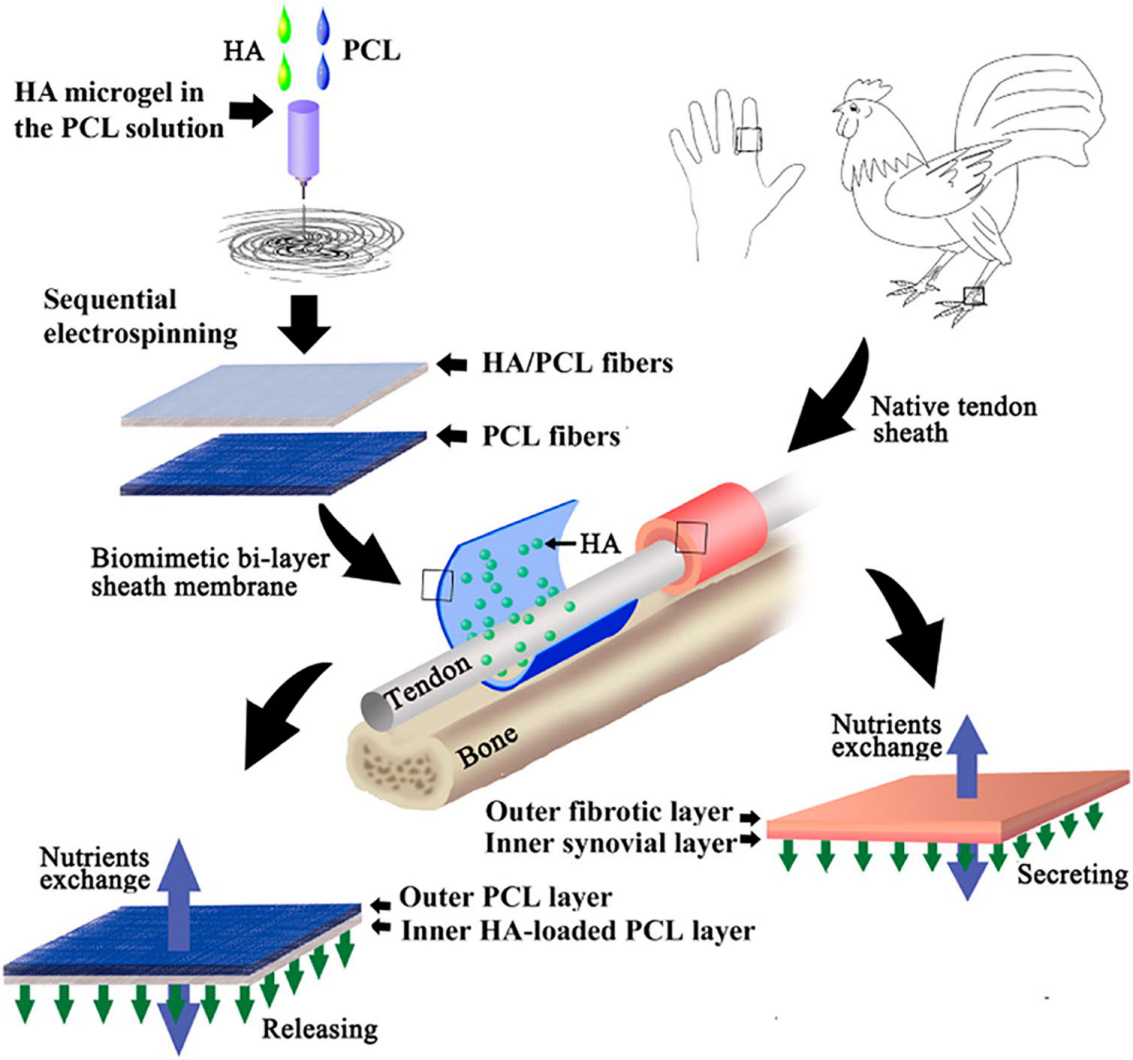
After formation of the HA/PCL membrane, it was applied to tendon. Adapted with permission from Liu et al. [40]

The tendon sheath is composed of an outer fibrotic layer and an inner synovial layer. The fibrotic layer acts as an effective biological barrier preventing foreign cells from invading, whereas the inner layer secretes synovial fluid to promote tendon gliding and nourish the tendon. Thus, Liu et al. [40] combined sequential and microgel electrospinning technologies, to fabricate a biomimetic bilayer sheath membrane, which consists of a HA–loaded poly(ε-caprolactone) (HA/PCL) membrane as the inner layer, and a PCL fibrous membrane as the outer layer (Fig. 4). Because PCL is a biodegradable semi-crystalline polyester, with good mechanical properties such as stability and flexibility, it is suitable for drug loading and tissue repair [41]. Meanwhile, the ability of PCL to prevent adhesion is influenced by its viscosity-average molecular weight (Mη), with research showing optimum results at Mη of 80,000 [42]. However, PCL is hydrophobic, highly rigid, and has long degradation time, these properties limit its single application. Consequently, the combination of PCL and HA, a hydrophilic material with high deformation and easy degradation, can improve its shortcomings. Besides the tensile strength of the membranes decrease with increasing HA ratio, which makes it easier to operate and allows better wrapping of the damaged zone. Meanwhile, the outer layer can serve as a physical barrier to reduce external healing without affecting the infiltration of nutrients because of its high length-diameter ratio and porosity.

Another biomimetic membrane called electrospun chitosan-grafted polycaprolactone (PCL-g-CS) nanofibrous membrane (NFM) was composed of the outer membrane of PCL NFM and the inner PCL membrane with covalently grafted CS molecules. This membrane has its special properties as well as good performance of bionics [43]. CS is a glucosamine formed by partial deacetylation of chitin, which is the main component of the exoskeleton of crustaceans [21]. It possesses considerable biocompatibility and bioactivity and the degradation rate can be adjusted according to deacetylation degree (DD). In addition, it can reduce adhesion by selectivity promoting the proliferation of epithelial and endothelial cells while suppressing that of fibroblasts, and has hemostatic effects. However, its anti-adhesion performance depends on its granular form, which allows it to spread rapidly and widely [44]. Therefore, grafting CS to the surface of PCL NFM can obtain a more accurate and rapid anti-adhesion effect. Besides the combination also optimizes the mechanical properties and the tissue separation ability. Meanwhile, it shows better selective permeability and has increased hydrophilicity and tensile strength when compared with PCL NFM. When compared with groups treated with Seprafilm, PCL, or phosphate-buffered saline (PBS) solution, there was a significant increase in the healed tendon mechanical strength in the group treated with the PCL-g-CS NFM.

Shalumon et al. [45] formed core-shell nanofibrous membranes (CSNMs) by combining the poly(ethylene glycol) (PEG)/PCL shell containing silver nanoparticles (Ag NPs) and HA/ibuprofen core to establish a more effective anti-adhesion barrier. Ibuprofen is a common clinical antipyretic, analgesic and anti-inflammatory drug. Therefore, its use can relieve postoperative pain, which may lead to higher compliance of the patient, and can reduce adhesion promoted by acute post-surgical inflammatory response. Applications of ibuprofen in tissue protection and regeneration have also been reported. However, its high concentrations *in vitro* and *in vivo* result in potentially cytotoxicity. Therefore, wrapping it in hydrogel can control its release to small doses, thereby reducing the adverse reactions. Ag NPs are one of the most popular materials in clinical applications. They can block cell function by inactivating proteins and enzymes in cell membranes and reduce the activity of matrix metalloproteinases (MMPs), thereby promoting wound healing. At the same time, they can induce the loss of K+ ions, thus damaging the cell membrane, and eventually leading to cell death. Consequently, they have effective antibacterial property against common infection-causing pathogens, including Gram-positive *S. aureus* and Gram-negative *E. coli*, without inducing drug resistance. When applied to membrane, the toxicity of Ag is very low. As a result, Ag NPs are embedded in the PCL/PEG shell. In addition, these CSNMs have excellent elasticity and flexibility to wrap damaged areas without tearing.

In summary, as an anti-adhesion barrier, the electrospun fiber membrane has advantages of simulating the structure of a natural tendon sheath, has an appropriate degradation rate, is easily functionalized, has certain mechanical strengths, has good biocompatibility, has a limited immune activity and has scaffold effect. However, its application still has many challenges. For example, the activity loss of therapeutically related substances due to the electric field force needs to be reduced, uniform distribution of therapeutic substances on the membrane needs to be realized, adverse effects of the substance need to be controlled and nanoparticles encapsulation efficiency needs to be improved. Furthermore, reduction in cost and simplification of the preparation process is required. Compared to hydrogel, the membrane is not easy to operate and cause the increase of tendon thickness which adversely affects the frequent sliding movement. Besides, despite the need for the appropriate mechanical tensile strength, the fiber membrane thickness, pore size and porosity affect cell infiltration. Overall, simulating the natural sheath hierarchy and biological functions is still a serious challenge in the field of materials science [40].

#### Absorbable films

Absorbable films are composed of different polymer materials that can be used as physical barriers to prevent adhesion. According to the properties of the constituent materials, these films can be divided into natural films and synthetic films. Natural membranes are compatible with biological tissue and have abundant sources but for some reasons they are the last choice for adhesion prevention. Synthetic films, which consist of many polymers, can be specially designed to degrade *in vivo* with an adjustable degradation rate and have good mechanical properties; particularly, they do not tear easily during suturing [21]. In addition, many polymers can reduce the inflammatory response, which has a significant impact on adhesion formation [46].

##### Natural films

Reconstituted type I collagen membrane is a kind of membrane produced by crosslinking collagen fibers under the action of the zero-length cross-linking agent 1-ethyl-3-(3-dimethylaminopropyl) carbodiimide (EDC) and *N*-hydroxysuccinimide (NHS) [47]. Due to its physical and chemical properties and structure being similar to natural tissue, it has good biological reaction and biocompatibility. This membrane also permits the penetration of growth factors and cytokines necessary for tendon healing [18] and is thin to suture easily. However, when the EDC concentration is higher than the upper limit of normal value, it will have negative effects on cell adhesion and proliferation, which lead to decreased cell activity and poor prognosis of the injured tissues. Meanwhile, the higher degree of crosslinking slows down the degradation rate of the membrane and adversely affects the mechanical properties, leading to the increase of fiber stiffness, and decreased ultimate tensile strength [47].

Integra is made of bovine collagen and shark cartilage glycosaminoglycan (GAG) to prevent adhesion in tendon healing [48]. GAG exists in mammalian cells extensively, for example, HA is one class of it. It plays an important role in various physiological and pathological processes including infections and inflammation. Exogenous sulfated glycans of various structures can downregulate inflammation processes, which is crucial to the post-operation adhesion of the tendon [49]. Therefore, Integra can significantly reduce inflammatory response and scar formation compared with pure collagen membrane. Besides, the microporous structure of Integra permits the penetration of essential growth factors and cytokines but blocks fibroblasts, thereby selectively inhibiting external healing and promoting internal healing. Concurrently, the membrane provides a scaffold for the growth of tendon cell to promote postoperative healing. This membrane is also biocompatible and will not affect the strength of healed tendon [48]. However, during surgical treatment, Integra was found to be large and difficult to suture [18]. Meanwhile, GAG is usually extracted from animals, thus, its performance cannot be guaranteed. It has complex molecular composition with exceptional structural diversity, which hinder its success in clinical trials [49].

Liu et al. [50] investigated the role of decellularized amnion as a physical barrier against postoperative tendon adhesion, and found that after enzymatic treatment, the acellular amniotic membranes reduced its antigenicity and thickness, but still maintained good biocompatibility, permeability, and degradability, whereas the integrity and smoothness of the membranes enabled it to act as a physical barrier against adhesion and a sheath for tendon slippage. The membrane also had anti-inflammatory properties and promoted tissue regeneration due to the release of active substances such as interleukin-1 receptor antagonists (IL-1 Ra). In addition, their scaffold function promotes collagen fiber alignment, thus optimizing tendon healing strength. However, the unilateral amniotic membrane is too thin to be injured in the process of tendon sliding. In order to solve this problem, the membrane needs to be cultured with a variety of cells to establish an appropriate thickness and become an ideal scaffold for anti-adhesion engineering.

##### Synthetic films

A porous poly l-lactide-co-ε-caprolactone (P(LA/CL)) membrane has been investigated by Sato et al. [51]. This membrane is absorbable, and the rate of degradation can be adjusted by changing its MW or the proportion of lactic acid. Additionally, its final degradation products are carbon dioxide and water. Therefore, it can be designed to cover the critical period of tendon healing without additional healing and damage to the tendon after it degrades. Besides, its high porosity allows the penetration of synovial fluid, which is an important nutrient source for inner tendons. Furthermore, it has a high tensile strength, which makes it hard to tear during operation.

Waterborne biodegradable polyurethane films (WBPU) combine PCL and polyurethane (PU) to neutralize the shortcomings of PCL [46]. PU consists of soft segment and hard segment forming a microphase-separated structure with excellent flexibility and mechanical properties [52]. This specific structure can also affect the rate of biodegradation. It allows PUs to change their surface composition in different environments, as more polar hard segments the interface when the environment is polar, in order to minimize the free energy of interface and promote *in vivo* degradation. Additionally, the anti-adhesion functions of PU are mainly dependent on the hard segment. Besides, the ion groups in WBPU make it hydrophilic compared with PU alone, and shows better immunosuppressive activity.

In summary, the materials of absorbable films discussed above are biocompatible, biodegradable, and can inhibit the inflammatory response to varying degrees. Therefore, they can be used effectively against tendon adhesion. On the downside, most of the available off-the-shelf films are single-function and are difficult to deal with in common complex situations and lesions. In addition, compared to water gel, the films are not easy to operate and their mechanical strength and degradation rate still need to be further studied.

## Discussion

Adhesion is manifested as proliferated fibrous tissues sticking to the nearby normal area [41]. Whilst acknowledging the improvements in surgical techniques and postoperative treatment plans, tendon adhesion remains a major challenge after tendon injury and triggers severe disability, reduced quality of life, expensive medical expenses, and declination of productivity. Therefore, preventing or reducing tendon adhesion is the present pivotal subject of focus in research.

Tendon adhesion occurs as the result of an imbalance between internal and external tendon healing mechanisms. Notably, intrinsic healing dominated by endotenon cells tends to promote tendon reconstruction and minimize tendon adhesion. External healing, on the other hand, primarily relies on inflammatory cascades and causes unorganized collagen deposition, thereby facilitates adhesion formation [48].

To better understand tendon healing, its pathological processes should be probed, for instance, the first inflammatory period spanning for about 1 week has a significant effect on the outcomes of healing. During the first 24 h, the dominant monocytes release colossal amounts of inflammatory cytokines which promote neovascularization and encourage the proliferation and directed migration of tenocytes [53]. At this point, collagen III synthesis begins which leads to the formation of adhesion [1]. Then, during the proliferation period, fibroblasts proliferate significantly and collagen synthesis of type-III reaches its peak [53]. Subsequently, remodeling period commences, and in the first 10 weeks called the consolidation period, cells decrease, repaired organization fibrosis gradually [6], and there is a high proportion of type-I collagen synthesis which is beneficial to the effective biomechanical properties of repaired tendons [1].

Besides the macroscopic level, discoveries at the molecular level are also thought to be the key events. Derby et al. found that the expression product of Reactive gene-1 enhances exogenous healing and deteriorates adhesion. At the same time, Loiselle et al. investigated a Smad3 protein and found that its overexpression could aggravate the adhesion [21]. MMPs were found to participate both in collagen degradation and collagen remodeling. Additionally, nitric oxide synthase influences the properties of neovascularization and the internal environment by promoting the synthesis of nitric oxide, and the formation of nerve fibers to transmit neuropeptides with signaling effects thereby regulate tendon healing. Substance p and calcitonin gene-related peptide (CGRP) promote inflammation and often reach a concentration peak in tissue during the proliferation period [53]. In addition, cytokines and growth factors have various effects on tendon healing and adhesion formation in every period of tissue repair, including Insulin-Like Growth Factor (IGF), Platelet-Derived Growth Factor (PDGF), Basic Fibroblastic Growth Factor (BFGF), Bone Morphogenetic Proteins (BMP), Transforming Growth Factor beta (TGF-β), and Vascular Endothelial Growth Factor (VEGF) [1]. For example, TGF-β promotes scar formation and tissue fibrosis [54].

Based on available information on adhesion formation, biomaterials often act as a physical barrier to isolate damaged tissue from contact with the surrounding tissues, thereby preventing migration of foreign fibroblasts and inhibiting exogenous healing. Additionally, a few of them are implicated in the regulation of molecular level and inflammatory response. Nearly all of these materials exhibit efficient biocompatibility, and they are biodegradable, notably, their rate of degradation can be adjusted according to clinical needs, implying that the tendon does not need another operation to remove the material after healing. Furthermore, a few of these biomaterials have certain mechanical strength that reduces the tensile load on the damaged tendon. Several materials can also be combined with active substances to slowly release functional molecules, in order to play a continuous role in the injury site, thereby improving the anti-adhesion effect of active substances, and simplifying clinical operation.

As mentioned above, many anti-adhesion materials have shown satisfactory and various anti-adhesion advantages, but there are still many challenges that need to be resolved including,

1. There is no uniform set of standards in this area of research. For example, in standard animal models, some researchers prefer chickens or rats while others prefer rabbits. In addition, various research methods were selected including two different treatments on the same animal and different groups of animals according to the treatment methods. At the same time, the selection of various parameters, including inflammatory indicators, biomechanical strength, and adhesion degree, are diverse. This makes it difficult for practitioners to determine and compare the test results.
2. The duration of the research is insufficient. Most of the existing studies stop at approximately the proliferation period of tendon healing when the material has not completely degraded. Therefore, it is impossible to know whether the material fully truncates the whole adhesion formation reaction or just delays the occurrence of the reaction. In addition, it remains unknown whether the non-degraded anti-adhesion barrier causes additional healing or influence the original healing process.
3. More deliberation needs to be given to the microstructure of the material. For example, the thickness, pore diameter and porosity of the membrane affects the space of the tendon sheath, tendon slipping ability, infiltration of cells and various functional molecules as the result of a bi-functional anti-adhesion scaffold studied by Liao et al. [34]
4. Lack of clinical practices. It should be remembered that in animal experiments, there are aspects different from humans [11], including anatomical characteristics, healing potentials, and resistance to infection, tolerance of foreign matter or immune responses, as well as compliance in the process of treatment, etc. Therefore, animal studies cannot be directly applied to humans and all types of material still need to be validated in a large sample size of strict and extensive comparative experiments.
5. Variations in tenocytes phenotype expression during tendon repair have not been extensively studied. Different anatomical and physiological characteristics of the tendon can lead to different cellular events after injury. For example, flexor tendon injury is initiated by the migration of fibroblasts from the tendon sheath into the tendon to initiate healing, whereas rotator cuff injury is initiated by the output of fibroblasts from the tendon to the bone surface [6]. At the same time, single cells undergo functional changes in different internal environments and thereby affecting adhesion formation. Macrophages, for example, undergo phenotypic transformation driven by specific conditions during tendon healing, thus performing different or even opposite functions. Therefore, further investigation might help optimize anti-adhesion strategies.
6. Ignorance of internal healing. Current interventions generally focus on inhibiting external healing mechanisms that reduce adhesion formation, but few studies have explored whether regulating endogenous mechanisms influence adhesion formation. Due to the inhibitory role of materials, they might often unknowingly inhibit endogenous healing thus affects the strength and speed of tendon healing. Besides, early initiation of endogenous mechanisms can promote early strengthening of tendon strength, which might be conducive to early postoperative exercise to reduce adhesion.

## Further perspectives

In this paper, we reviewed the application characteristics of three kinds of biomaterials, which have become popular for the past decade in the fight against tendon adhesion. Together with these results and the enthusiasm of scientists, it is believed that with the continuous innovation and attempts of researchers, additional novel materials will be invented in this field. Therefore, our recommendation for future research includes,

1. Given that many successful materials in animal studies have disappeared from the anti-tendon adhesion library for the lack of further research to facilitate their entry into clinical practice, whereas silk continues to undergo innovations and application since its first use in 1914. Therefore, the future direction of materials is bound to develop based on low price, wide source of materials, convenient production process, and high practical operability.
2. By chemical modification or through physical blending, new materials that combine the unique advantages of the three kinds of biomaterials we mentioned above can be prepared. For example, exaggerated, we can build a type of material exhibiting the characteristics of the hydrogel, which means it can be injected into the damaged area with less trauma, and the same time when it has enters the body, according to the specific physical and chemical properties of the damaged place or internal environment change, the material could accurately locate and then rapidly form a membrane with stent performance. Additionally, in the process of tendon healing, it can gradually degrade at an appropriate rate to develop the ideal anti-adhesion barrier.
3. Develop the carrier functions of materials, such as loading multiple drugs, multiple functional molecules like cytokines, growth factors, or antibodies, to produce continuous or synergistic effects of active substances. Stem cells can also be loaded to promote the differentiation and proliferation of desired cell types, thus facilitating the healing of specific tissues. In addition, appropriate active substances should be selected for loading according to different healing mechanisms of different injury sites.
4. It might be more helpful to confine drugs or other functional molecules to local areas, which can enhance the local effects of functional substances, and avoid the possible side effects of substances. In addition, adjusting the drug release dose according to the treatment stage or releasing drugs layer by layer is related to this purpose.
5. The natural tendon sheath supports and protects the structure of the tendon, and secretes synovial fluid to produce nutrition and assist in the sliding of the tendon. Due to the complexity of its function and structure, the development of a bionic tendon sheath as an ideal anti-adhesion barrier has shown greater potential.
6. There should be a choice of a more specific functional molecule that promotes endogenous mechanisms as well as inhibitors of exogenous factors and thereby better reduce tendon adhesion.

## Data Availability

The dataset supporting the conclusions of this article is included with the article.
